# Long-term allograft and patient outcomes of kidney transplant recipients with and without incident cancer – a population cohort study

**DOI:** 10.18632/oncotarget.20781

**Published:** 2017-09-08

**Authors:** Wai H. Lim, Sunil V. Badve, Germaine Wong

**Affiliations:** ^1^ Department of Renal Medicine, Sir Charles Gairdner Hospital, Nedlands, Australia; ^2^ School of Medicine and Pharmacology, University of Western Australia, Perth, Australia; ^3^ Australia and New Zealand Dialysis and Transplant Registry, Adelaide, Australia; ^4^ Department of Renal Medicine, St. George Hospital, Sydney, Australia; ^5^ Renal and Metabolic Division, The George Institute for Global Health, Sydney, Australia; ^6^ University of New South Wales Medicine, Sydney, Australia; ^7^ Centre for Transplant and Renal Research, Westmead Hospital, Sydney, Australia; ^8^ Centre for Kidney Research, The Children's Hospital at Westmead, Sydney, Australia; ^9^ Sydney School of Public Health, University of Sydney, Sydney, Australia

**Keywords:** registry, cancer, kidney transplantation, death

## Abstract

The excess risk for cancer in kidney transplant recipients is substantial, but the allograft and patient survivals after cancer development are under-studied. This is a population-based cohort study of all primary live and deceased donor kidney transplant recipients in Australia and New Zealand between 1990-2012. The risks of overall graft loss and death with a functioning graft in kidney transplant recipients with and without incident cancer were determined using adjusted Cox regression analysis, with incident cancer considered as a time-varying covariate in the models. In those with incident cancer, types and cancer stage at diagnoses were reported. Of 12,545 transplant recipients followed for a median of 6.9 years (91,380 patient-years), 1184 (9.4%) developed incident cancers at a median of 5.8 years post-transplant. Digestive, kidney and urinary tract cancers were the most common cancer types, although digestive and respiratory tract cancers were more aggressive, with 40% reported as advanced cancers at time of cancer diagnosis. Cancer-related deaths accounted for approximately 80% of recipients with a prior cancer history. Compared with recipients with no prior cancer, the adjusted hazard ratios (HR) for overall graft loss and death with functioning graft were 4.34 (95%CI 3.90, 4.82; p<0.001) and 9.53 (95%CI 8.30, 10.95; <0.001) among those with a prior cancer. Incident cancer after kidney transplantation is a significant risk factor for death with a functioning graft, with the majority of deaths attributed to cancer. A greater understanding of the barriers to screening and treatment approaches following cancer diagnosis may lead to improve survival in kidney transplant recipients with cancer.

## INTRODUCTION

Cancer is one the leading causes of death after kidney transplantation worldwide [1, 2]. In Australia, cancer death has surpassed deaths attributed to cardiovascular disease (CVD) after kidney transplantation, likely reflecting a combination of improved preventive strategies and more aggressive management of CVD risk factors [3]. Recipients of kidney transplants have up to a 3-fold greater risk of incident cancers compared to age- and gender-matched general population, with both the cumulative burden of long-term immunosuppressive therapy and chronic viral infections likely to have a crucial role in carcinogenesis after transplantation [4, 5]. Following cancer diagnosis, kidney transplant recipients are more likely to experience premature death compared to age-matched general population with cancer, possibly related to more aggressive disease at presentation and/or inadequate treatment because of the likelihood of concomitant comorbidities [5, 6].

The prognosis and pattern of death in kidney transplant recipients who have developed incident cancer varies according to the type and severity of cancer at presentation. For example, in patients who have developed melanoma after kidney transplantation, 42% of deaths were directly attributed to cancer, suggesting that over 50% of deaths may not be directly related to cancer [7]. Moreover, it remains unclear whether similar patterns of death occur in recipients who have developed incident cancers at other sites. A greater understanding of the mortality patterns in recipients who have developed incident cancer may help clinicians and researchers identify potential modifiable factors that may contribute to the poorer prognosis in this population. The aim of this study was to compare the pattern of allograft loss, cancer-specific and all-cause mortality in kidney transplant recipients without and with incident cancer using data from the Australia and New Zealand dialysis and transplant (ANZDATA) registry.

## RESULTS

### Study population

There were 12,545 recipients included in this study, of which 11,361 (90.6%) did not develop incident cancer, 1184 (9.4%) developed incident cancer prior to graft loss, with a median (interquartile range [IQR]) time to cancer development of 5.8 (7.2) years. Baseline characteristics of the study population are shown in Table 1. The median (IQR) patient-follow-up period for all recipients was 7.2 (9.2) years resulting in 103,265 patient-years of follow-up, with longer median (IQR) follow-up period for recipients who developed cancer (10.8 [8.8] years with 12,775 patient-years of follow-up).

**Table 1:** Baseline characteristic of live- and deceased donor kidney transplant recipients stratified by absence and presence of incident cancer between 1990-2012 (n = 12,545)

	No cancer (n=11,361)	Incident cancer (n=1184)	p-value
**Demographics**			
Age (years, mean±SD)	43.4±15.6	48.6±13.7	<0.001
Male (n, %)	7032 (61.9)	693 (58.5)	0.023
Race (n, %)			<0.001
Caucasian	9137 (80.4)	1053 (88.9)	
Indigenous	970 (8.5)	49 (4.2)	
Others	1254 (11.1)	82 (6.9)	
Coronary artery disease (n, %)			
Peripheral vascular disease (n, %)	1005 (9.1)	102 (9.1)	0.952
Cerebrovascular disease (n, %)	539 (4.9)	54 (4.8)	0.906
Body mass index (kg/m^2^, mean±SD)	329 (3.0)	32 (2.8)	0.813
Waiting time (years, mean±SD)	25.2±5.4	24.9±4.7	0.089
Diabetes (n, %)	2.5±2.5	2.5±2.3	0.233
Smoker (n, %)	1516 (13.3)	142 (12.0)	0.192
Non-smoker	6431 (59.8)	522 (51.0)	<0.001
Former smoker	3117 (29.0)	378 (36.9)	
Current smoker	1205 (11.2)	124 (12.1)	
Cause of ESKD (n, %)			
Glomerulonephritis	4868 (42.8)	517 (43.7)	<0.001
Cystic	1613 (14.2)	199 (16.8)	
Diabetes	1001 (8.8)	76 (6.4)	
Vascular	476 (4.2)	58 (4.9)	
Analgesic nephropathy	146 (1.3)	44 (3.7)	
Others	3257 (28.7)	290 (24.5)	
**Donor characteristics**			
Age (years, mean±SD)			
Type (n, %)	44.7±15.6	42.4±16.1	<0.001
Live-donor	4314 (38.1)	304 (25.9)	<0.001
Deceased donor	7012 (61.9)	868 (74.1)	
ABO-incompatible (n, %)			
**Immunology/Transplant**	178 (1.6)	5 (0.4)	0.002
HLA-ABDR mismatches (n, %)			
0	645 (5.7)	65 (5.5)	<0.001
1-2	3754 (33.3)	446 (37.9)	
3-6	6962 (61.0)	665 (56.6)	
Peak PRA >50% (n, %)			
Ischaemic time (hours, mean±SD)	992 (8.8)	128 (10.8)	0.001
Induction (n, %)	9.8±7.2	12.1±7.4	<0.001
Transplant era (n, %)	5309 (46.7)	284 (24.0)	<0.001
1990-1993	1355 (11.9)	322 (27.2)	<0.001
1994-1997	1462 (12.9)	275 (23.2)	
1998-2001	1729 (15.2)	240 (20.3)	
2002-2005	2073 (18.3)	206 (17.4)	
2006-2009	2479 (21.8)	105 (8.9)	
2010-2012	2263 (19.9)	36 (3.0)	
Initial immunosuppression (n, %)			
	9488 (95.7)	819 (95.7)	0.975
Prednisolone	336 (3.4)	20 (2.3)	
CNI	5622 (56.7)	662 (77.3)	<0.001
None	3961 (39.9)	174 (20.4)	
Cyclosporin	630 (6.4)	65 (7.6)	<0.001
Tacrolimus	7856 (79.2)	537 (62.7)	
Anti-metabolite	1433 (14.4)	254 (29.7)	
None			
MMF/myfortic			
Azathioprine			
Outcomes (n, %)			
Overall graft loss	3535 (31.1)	714 (60.3)	<0.001
Death-censored graft loss	2374 (20.9)	163 (13.8)	<0.001
Death with functioning graft	1161 (10.2)	552 (46.6)	<0.001
All-cause mortality	2103 (18.5)	612 (51.7)	<0.001

Data expressed as number (proportion) or as mean ± SD. ESKD – end-stage kidney disease, HLA – human leukocyte antigen, PRA – panel reactive antibody, CNI – calcineurin-inhibitor, MMF – mycophenolate mofetil.

Recipients who developed cancers were older, more likely to have a smoking history and were more likely to have received deceased donor transplants. The burden of comorbidities was similar across recipients with and without incident cancers. The incidences of site-specific cancer types are shown in Supplementary Figure 1. In recipients who have developed cancer (n=1184), digestive cancers were the most common type of cancer (n=228, 19.3%), followed by skin cancers (n=164, 13.9%), kidney/urinary tract cancers (n=136, 11.5%), female genital tract cancer (n=115, 9.7%), haematological cancers (n=100, 8.4%) and respiratory cancers (n=99, 8.4%).

### Associations between incident cancer, overall graft loss and death censored graft loss

**Figure 1:**
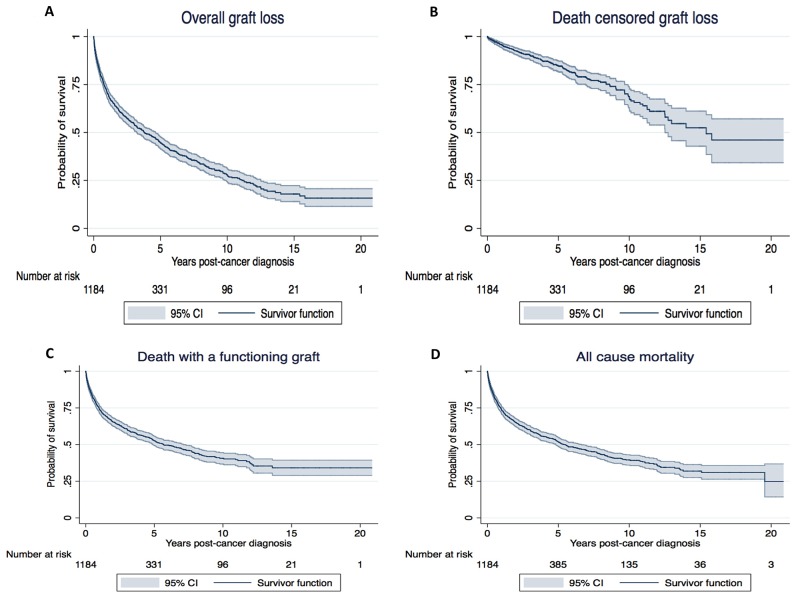
Kaplan Meier survival curves for overall graft loss (**A**), death censored graft loss (**B**), death with a functioning graft (**C**) and all-cause mortality (**D**) for recipients who have developed incident cancer after kidney transplantation. The “x” axis represents time from cancer diagnosis and “y” axis the probability of survival.

Following cancer diagnosis, the overall graft survivals at 1, 5 and 10 years for recipients who have developed incident cancer were 70.8% (95% confidence intervals [95%CI] 68.1, 73.4), 44.7% (95%CI 41.6, 47.7), and 27.9% (95%CI 24.5, 31.4), respectively (Figure 1A). Compared to recipients without incident cancer, the adjusted hazard ratio (HR) of recipients who have developed incident cancer was 4.34 (95%CI 3.90, 4.82; p<0.001) for overall graft loss. Other significant covariates are shown in Table 2.

**Table 2:** The adjusted hazard ratios for overall graft loss, death-censored graft loss and death with a functioning graft in recipients with and without incident cancers

	Overall graft lossAdjusted HR (95%CI)	Death censored graft lossAdjusted HR (95%CI)	Death with a functioning graftAdjusted HR (95%CI)
Incident cancer			
None	1.00	1.00	1.00
Yes	4.34 (3.90, 4.82)	1.43 (1.16, 1.77)	9.53 (8.30, 10.95)
Coronary artery disease	1.22 (1.08, 1.39)	-	1.23 (1.04, 1.46)
Peripheral vascular disease	1.31 (1.12, 1.54)	-	1.36 (1.10, 1.68)
Cerebrovascular disease	1.44 (1.19, 1.74)	-	1.53 (1.20, 1.95)
Age (per year increase)	1.00 (1.00, 1.01)	0.98 (0.98, 0.99)	1.06 (1.05, 1.06)
HLA-mismatch (per mismatch)	1.08 (1.06, 1.11)	1.11 (1.07, 1.14)	-
Donor type:			
Live-donor	-	1.00	1.00
Deceased-donor	-	1.20 (1.00, 1.45)	1.04 (0.82, 1.31)
Ischaemic time (per hour increase)	1.015 (1.009, 1.020)	1.00 (0.99, 1.02)	1.01 (1.00, 1.03)
Race: Caucasian	1.00	1.00	1.00
Indigenous	1.78 (1.58, 2.02)	1.95 (1.67, 2.27)	1.62 (1.32, 2.00)
Others	0.96 (0.84, 1.09)	0.98 (0.83, 1.16)	0.98 (0.79, 1.22)
Waiting time (per year increase)	1.05 (1.03, 1.06)	1.04 (1.01, 1.06)	1.09 (1.06, 1.11)
Diabetes	1.26 (1.03, 1.54)	1.20 (0.89, 1.60)	1.43 (1.09, 1.88)
Smoking:			
Non-smoker	1.00	1.00	1.00
Former smoker	1.12 (1.03, 1.22)	1.18 (1.05, 1.32)	1.13 (0.99, 1.29)
Current smoker	1.46 (1.31, 1.63)	1.53 (1.33, 1.75)	1.62 (1.35, 1.94)
Donor age (per year increase)	1.01 (1.01, 1.02)	1.02 (1.02, 1.03)	1.01 (1.00, 1.01)
Peak PRA:			
0-10%	1.00	1.00	1.00
11-50%	1.03 (0.94, 1.14)	0.96 (0.84, 1.09)	1.10 (0.95, 1.28)
51-75%	1.15 (0.96, 1.36)	1.06 (0.84, 1.33)	1.40 (1.07, 1.83)
>75%	1.28 (1.10, 1.49)	1.33 (1.09, 1.62)	1.32 (1.05, 1.67)

Data expressed as adjusted hazard ratio (HR) and 95% confidence intervals (95%CI). DCGL – death censored graft loss, DFG – death with a functioning graft, BMI – body mass index, HLA – human leukocyte antigen, PRA – panel reactive antibody.

Following cancer diagnosis, the overall death censored graft survivals at 1, 5 and 10 years for recipients who have developed incident cancer were 95.9% (95%CI 94.4, 96.9), 84.9% (95%CI 81.9, 87.4), and 68.7% (95%CI 62.9, 73.8), respectively (Figure 1B). Compared to recipients without incident cancer, the adjusted HR for death censored graft loss (DCGL) in recipients who have developed incident cancer was 1.44 (95%CI 1.16, 1.77; p=0.001).

Table 3 shows the causes of graft loss in kidney transplant recipients with and without incident cancer. The predominant cause of graft loss in recipients who have developed incident cancer was death with a functioning graft (77%); whereas in recipients who did not develop cancer, chronic allograft nephropathy/interstitial fibrosis and tubular atrophy (CAN/IFTA, 37%) and death with a functioning graft (33%) contributed equally to overall graft loss. Acute rejection was the cause of graft loss in 1.9% and 6.5% of recipients with and without incident cancer, respectively. If death with a functioning graft was excluded as a cause of graft loss, the incidence of rejection-related death-censored graft loss was similar between kidney transplant recipients with and without incident cancers (9% vs. 10%, respectively, p=0.9).

**Table 3:** Causes of graft loss, death with a functioning graft and all-cause mortality in kidney transplant recipients with and without incident cancer

Causes of graft loss	No cancer (n=3535)	Incident cancer (n=714)
**Death**	1161 (32.8)	552 (77.3)
**Rejection**	213 (6.0)	3 (0.4)
**CAN/IFTA**	1300 (36.8)	113 (15.9)
**BKVAN**	25 (0.7)	4 (0.6)
**Donor cancer**	7 (0.2)	2 (0.3)
**GN**	207 (5.9)	10 (1.4)
**Cancer**		
** Malignancy invading graft**	2 (0.1)	11 (1.5)
** Withdrawal IS (rejection)**	2 (0.1)	10 (1.4)
**Renal vascular complications**	192 (5.4)	1 (0.1)
**Non-compliance**	124 (3.5)	4 (0.6)
**Infection**	34 (0.9)	3 (0.4)
**Withdrawal infection (rejection)**	14 (0.4)	1 (0.1)
**Others**	254 (7.3)	0 (0.0)
**Causes of death with functioning graft**	**No cancer (n=1161)**	**Incident cancer (n=552)**
**Cancer**	0 (0.0)	437 (79.1)
**Withdrawal**		
** Cancer**	0 (0.0)	8 (1.4)
** Comorbid vascular**	8 (0.7)	0 (0.02 (0.4)
** Psychosocial**	15 (1.3)	
**Cardiac**		
** Cardiac arrest**	157 (13.6)	12 (2.1)
** Myocardial ischaemia**	238 (20.6)	15 (2.6)
** Cardiac failure**	41 (3.5)	2 (0.9)
**CVA**	96 (8.3)	12 (2.1)
**Pulmonary embolus**	17 (1.5)	0 (0.0)
**Septicaemia (no source)**		
** Bacterial (include UTI)**	75 (6.5)	7 (1.2)
** Fungal**	8 (0.7)	1 (0.2)
** Viral**	10 (0.9)	1 (0.2)
** Others**	31 (2.3)	5 (0.9)
**Cachexia**	12 (1.0)	1 (0.2)
**Urinary tract infection**	11 (0.9)	0 (0.0)
**CNS infections**	24 (2.1)	1 (0.2)
**Liver infection**	4 (0.3)	1 (0.2)
**Lung infections**	134 (11.5)	9 (1.5)
**Unknown**	46 (4.0)	6 (1.1)
**Haemorrhage/blood loss**	18 (1.6)	4 (0.7)
**Others**	216 (18.7)	28 (5.0)
**Causes of all-cause mortality**	**No cancer (n=2103)**	**Incident cancer (n=612)**
**Cancer**	9 (0.4)	445 (72.7)
**Withdrawal**		
**Cancer**	4 (0.2)	15 (2.5)
**Comorbid vascular**	53 (2.5)	6 (1.0)
**Psychosocial**	71 (3.4)	5 (0.8)
**Cardiac**		
**Cardiac arrest**	318 (15.1)	21 (3.4)
**Myocardial ischaemia**	400 (19.0)	22 (3.5)
**Cardiac failure**	81 (3.8)	9 (1.5)
**CVA**	148 (7.0)	16 (2.6)
**Pulmonary embolus**	21 (1.0)	0 (0.0)
**Septicaemia (no source)**		
**Bacterial (include UTI)**	145 (6.9)	8 (1.3)
**Fungal**	14 (0.7)	1 (0.2)
**Viral**	16 (0.8)	2 (0.4)
**Others**	41 (1.9)	8 (1.3)
**Cachexia**	19 (0.9)	1 (0.2)
**Urinary tract infection**	12 (0.6)	1 (0.2)
**CNS infections**	32 (1.5)	1 (0.2)
**Liver infection**	6 (0.3)	1 (0.2)
**Lung infections**	191 (9.1)	9 (1.5)
**Unknown**	65 (3.1)	7 (1.1)
**Haemorrhage/blood loss**	33 (1.6)	2 (0.3)
**Others**	424 (20.2)	32 (5.1)

CAN/IFTA – chronic allograft nephropathy/interstitial fibrosis, BKVAN – BK viral allograft nephropathy, IS – immunosuppression, GN – glomerulonephritis, CVA – cerebrovascular accident, UTI – urinary tract infection, CNS – central nervous system.

### Association between incident cancer and death with a functioning graft

Following cancer diagnosis, the overall death with a functioning graft survivals at 1, 5 and 10 years for recipients who have developed incident cancer were 74.0% (95%CI 71.3, 76.4), 52.6% (95%CI 49.4, 55.7), and 40.6% (95%CI 36.8, 44.5), respectively (Figure 1C). Compared to recipients without incident cancer, the adjusted HR of recipients with incident cancer was 9.53 (95%CI 8.30, 10.95; <0.001) for death with a functioning graft. Other significant covariates are shown in Table 2.

Table 3 shows the causes of death with a functioning graft in kidney transplant recipients with and without incident cancer. Over 80% of deaths with a functioning graft in recipients with cancer were attributed to cancer or withdrawal as a result of cancer. CVD (38%) and infection (25%) were the two leading causes of death with a functioning graft in recipients without cancer, compared to 6% and 4% respectively in recipients with incident cancer.

### Association between incident cancer and all-cause mortality

Following cancer diagnosis, the overall patient survivals at 1, 5 and 10 years for recipients who have developed incident cancer were 73.4% (95%CI 70.8, 75.9), 51.7% (95%CI 48.6, 54.7), and 39.5% (95%CI 36.0, 43.0), respectively (Figure 1D). Compared to recipients without incident cancer, the adjusted HR of recipients who have developed incident cancer was 5.83 (95%CI 5.17, 6.59; p<0.001) for all-cause mortality. Table 3 shows the cause-specific mortality in kidney transplant recipients with and without incident cancer. Cancer (including withdrawal) was the predominant cause of mortality for recipients with cancer, contributing 75% of overall mortality in recipients who have developed incident cancer. In recipients without cancer, CVD and infection causes contributed to 38% and 22% of overall mortality, compared to 8% and 5%, respectively for recipients who have developed incident cancer.

### Site-specific cancer types: association with death with a functioning graft

**Figure 2:**
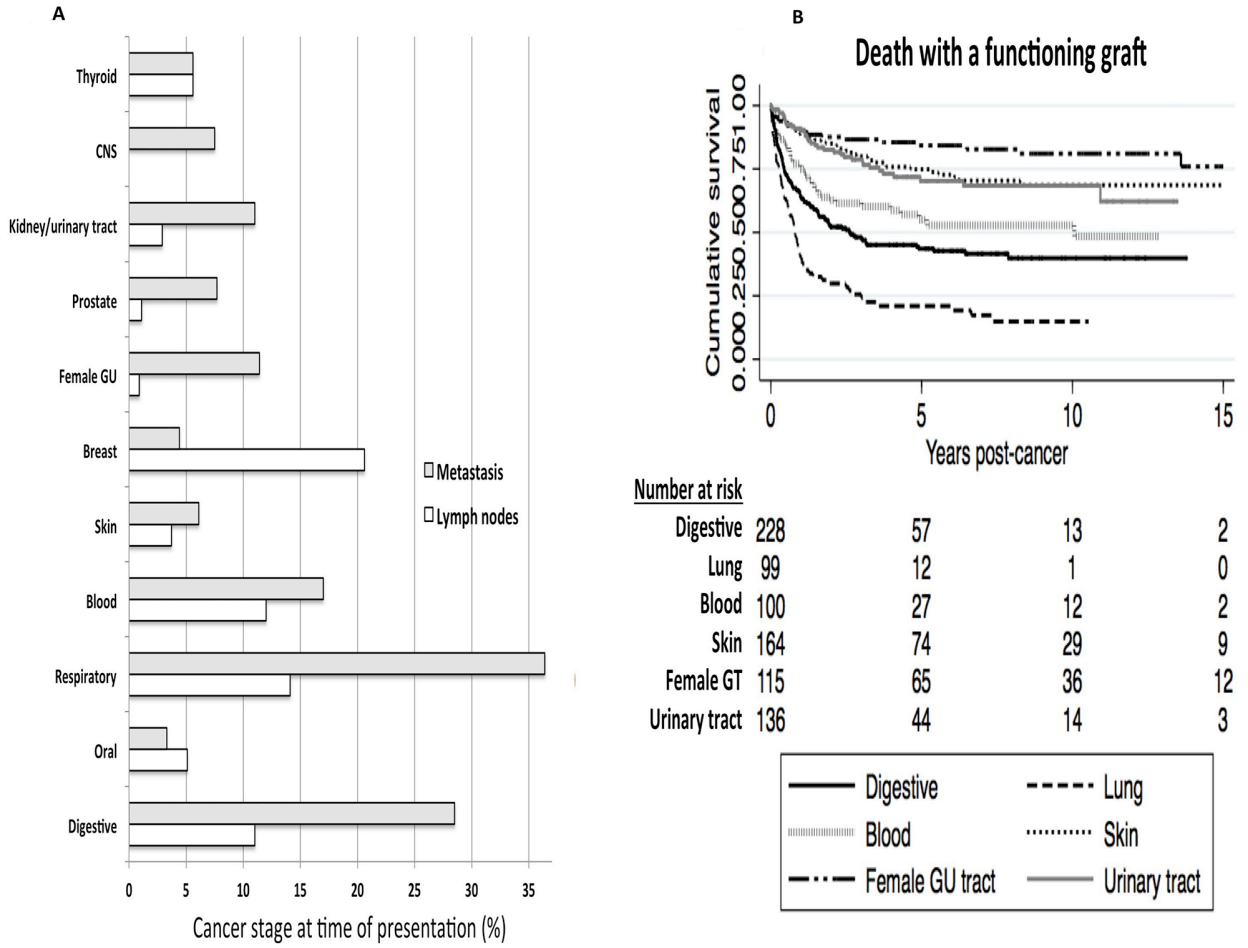
Site-specific cancer types The proportion of site-specific cancers with advanced stage disease (i.e. lymph nodes involvement or metastatic disease) at time of cancer diagnosis. Lymph node involvement represented by hollow bars and metastatic disease represented by shaded bars. CNS – central nervous system, female GT – female genital tract (**A**). Kaplan Meier survival curves with number at risk tables for death with functioning graft according to the six common site-specific cancers. Log-rank p<0.01 (**B**).

Supplementary Table 1 shows the characteristics of the common cancers, including sites, timing of cancer diagnosis, cancer stage, and the incidence of overall graft loss and death with a functioning graft. In recipients who have developed incident cancer, the majority of the cancers occurred at least 5-years post-transplant, except for skin and female genital tract cancers where almost 40% occurred within the first 3-years after transplant. Cancer is the principal cause of death with a functioning graft or all-cause mortality for all site-specific cancer types. Of the different cancer types, respiratory and digestive cancers were more aggressive at presentation, with over 40% reported as advanced cancers (i.e. involvement of lymph nodes or metastatic disease) at time of cancer diagnosis (Figure 2A). Kaplan Meier survival curves for death with a functioning graft according to site-specific cancer types are shown in Figure 2B. Of all recipients who have developed incident cancer, those with respiratory and digestive cancers were more likely to die with a functioning graft.

### Sensitivity analysis

To exclude reverse causality, recipients who had developed incident cancer within the first 2 years after transplantation were excluded (n=250). Compared to recipients without cancer, the adjusted HRs of recipients who had developed incident cancer were 4.37 (95%CI 3.86, 4.96; p<0.001) for overall graft loss, 1.43 (95%CI 1.12, 1.84; p=0.005) for DCGL, 9.68 (95%CI 8.23, 11.38; p<0.001) for death with a functioning graft and 5.78 (95%CI 5.02, 6.66; p<0.001) for all-cause mortality.

## DISCUSSION

Kidney transplant recipients who have developed incident cancer before graft loss have over a 9-times risk of death with a functioning graft compared to those without cancer, with 26% and 47% of recipients with incident cancer died with a functioning graft at 1 and 5-years post-cancer diagnosis. In addition, over 50% of recipients who have developed incident cancer lost their grafts within 5-years post-cancer diagnosis, with an overwhelming excess of graft loss attributed to cancer-related deaths with a functioning graft. Of all cancer types, digestive and respiratory cancers were associated with poorer outcomes, reflecting more advanced stage disease at the time of cancer diagnoses.

Cancer is common after kidney transplantation, with cumulative incidence of cancer in primary kidney transplant recipients in excess of 60% after 20 years post-transplant [8]. Epidemiological studies have consistently shown that the incidences of multiple cancers, particularly viral- and immune-mediated cancers are significantly greater in kidney transplant recipients compared to the age- and gender-matched general population. A population-based cohort study using record linkage between ANZDATA Registry and the Australian National Cancer Statistics Clearing House showed that after kidney transplantation, cancer incidences at 25 sites were significantly increased compared to the general population, with standardised incidence rations (SIR) exceeding 3-fold for cancer with known or presumed viral etiology [4]. Similarly, a linkage study using United States data of 202,195 kidney transplant recipients showed that the incidences of most cancers were increased after kidney transplantation, with the incidences of viral-related (e.g. lymphoma) and immune-related cancers (e.g. melanoma) higher during periods of graft function compared to following graft loss, but the inverse was observed for ESKD-related cancers (e.g. renal cell cancers) [5]. Other registries from around the world have shown similar patterns of incident cancer after transplantation [6, 9, 10]. There are geographical differences in the incidence of site-specific cancers, possibly related to dissimilar genetic, environmental exposure and/or viral patterns between countries. In this study, we have shown that digestive, skin and kidney/urinary tract cancers were the most common incident cancer types occurring post-kidney transplantation, reflecting that the pathogenesis of cancer risk is likely to be multifactorial. The majority of the cancers after transplantation except skin and female genital tract cancers tend to occur at least 5-years post-transplant suggesting that the cumulative exposure of immunosuppression is critical in the pathogenesis of the majority of these cancers.

Patient survival after cancer diagnosis is significantly poorer in kidney transplant recipients compared to the general population. Data from the ANZDATA registry showed that mortality is at least 40% greater in recipients with breast cancer or colorectal cancers compared to the general population [11]. In the Scientific Registry of Transplant Recipients (SRTR) analysis, lung cancers were more likely to be at an advanced stage at presentation in kidney transplant recipients compared to the general population, suggesting the possibility of accelerated cancer growth in recipients as a result of chronic immunosuppression [5]. However, the lack of effective screening for lung cancer may have contributed to these differences. Data from the Israel Penn Registry of 635 adult kidney transplant recipients showed that cancer stage-specific survivals for common cancers, including colorectal, lung and renal cell cancers were significantly poorer compared to the general population [12], suggesting that factors other than more advanced disease at presentation are likely to contribute to the survival differences. In a nested case-controlled study of 12,805 kidney transplant recipients registered in the Netherlands Organ Transplant Registry, the median patient survival of recipients who developed invasive cancer is 4-times lower than age- and gender-matched recipients without invasive cancer (2 vs. 8 years, p<0.01), with over 80% of those with cancer died with a functioning graft that was directly attributed to cancer after a median of 8 years [13]. Similar to these cohort studies, our study has shown that over 40% of lung and digestive cancers were at advanced stages at presentation, but ANZDATA registry do not collect data on the uptake of cancer screening in kidney transplant recipients. There may be poorer uptake of routine cancer screening among kidney transplant recipients, which may in part attributed to the perception of patients and/or clinicians that cancer screening may not be cost-effective because the expected remaining lifetime of some transplant recipients is shorter than the time required to develop cancer [14]. In addition, the lack of validation of routine screening test in kidney transplant recipients and the perception by some clinicians of the inability of standard cancer treatment to alter cancer prognosis may be other factors contributing to poorer survival in kidney transplant recipients who have developed incident cancer [15–18]. The likelihood of a greater burden of other comorbidities, suboptimal kidney function, potential drug interactions, potential adverse events or reluctance to reduce the intensity of overall immunosuppression (for fear of graft rejection/failure) may influence the decisions of recipients and/or clinicians against pursuing aggressive treatment options or receive an ‘inadequate’ amount of chemotherapy treatment. For example, certain biological agents such as interleukin-2 therapy for metastatic renal cell cancer would be deemed unsuitable in kidney transplant recipients with functioning grafts due to the likelihood of inducing acute rejection with treatment [19]. In our study, the proportion of deaths attributed to withdrawal is relatively low suggesting indirectly that recipients with cancer are actively considering treatment. Nevertheless, as cancer-related deaths remain the predominant cause of mortality in kidney transplant recipients who have developed cancer, a greater understanding of the disease and patient-level factors contributing to mortality is urgently needed.

Despite experiencing a higher risk of DCGL in recipients who have developed incident cancer compared to those without, it is important to point out that 9% of DCGL in recipients who had developed cancer (vs. 10% in recipients without cancer) was attributed to acute rejection (majority as a result of immunosuppression withdrawal), suggesting that the concern over practices of reducing transplant-specific immunosuppression in inducing excess acute rejection resulting in graft loss may be unsubstantiated, although this observation is likely to be affected by the competing risk of premature mortality.

This study has a number of limitations. Selection bias is likely to exist because there may be systematic differences in the management of recipients who have developed cancer after transplant. There are likely to be unmeasured residual confounders such as the severity of comorbidities, aggressiveness of cancer types, differences in the intensity of immunosuppression (including the variation in the practice of reducing immunosuppression following cancer diagnosis) and adherence and responses to cancer treatments, which are not collected by ANZDATA registry but may have modified the association between cancer and outcomes.

Understanding the association between incident cancer and outcomes and the pattern of mortality are crucial in the long-term clinical management of kidney transplant recipients. Our findings are consistent with published literature showing that kidney transplant recipients who have developed cancer after transplant are more likely to die compared to those without cancer, with over 80% of deaths attributed to cancer. Given that a large majority of cancers that occur after transplant can be potentially detected by routine or targeted cancer screening methods (e.g. faecal occult blood test for colorectal cancers, Paps smear for cervical cancer, ultrasound surveillance for renal cell cancer), adherence to these screening guidelines should be encouraged. However, future studies evaluating the test performances of the routine cancer screening methods in kidney transplant recipients, understanding potential barriers to routine cancer screening, evaluating the cost-effectiveness of targeted screening as well as scrutinizing the reasons for the increased risk of mortality in kidney transplant recipients who have developed cancer are urgently needed. There has been considerable debate with regards to cancer screening and/or aggressive treatment of cancers in patients with ESKD because of the anticipated shortened patient survival and potential competing risk of CVD deaths in this population. Given that the majority of the kidney transplant recipients have better expected survival compared to ESKD patients on maintenance dialysis and the large proportion of deaths following cancer diagnosis is attributed to cancer, cancer screening and appropriate treatment following cancer diagnosis is probably warranted for kidney transplant recipients who develop cancer post-transplant.

## CONCLUSION

Incident cancer after kidney transplantation is a significant risk factor for death with a functioning graft and all-cause mortality, with the majority of deaths attributed to cancer. Digestive, respiratory and urinary tract cancers are the most frequent cancer types, often at an advanced stage at presentation. Strategies to improve cancer surveillance and a greater understanding of the barriers to screening and treatment approaches following cancer diagnosis may lead to improve survival in kidney transplant recipients with cancer.

## MATERIALS AND METHODS

### Study population

Primary live and deceased donor kidney transplant recipients in Australia and New Zealand between 1990-2012 were included in the analyses. Recipients of multiple organ grafts, those who have received prior grafts and those with a history of cancer at anytime prior to transplantation (except for non-melanoma skin cancers [NMSC]) were excluded. Incident cancers included all cancers except NMSC, pre-malignant or in-situ lesions. The clinical and research activities being reported are consistent with the Principles of the Declaration of Istanbul as outlined in the ’Declaration of Istanbul on Organ Trafficking and Transplant Tourism’.

### Exposure factor

Recipients were categorised according to whether they had developed incident cancer before graft loss. Recipients who had developed cancer after graft loss (n=219), recipients who were recorded as developing incident cancer on the day of graft loss (n=12) and recipients without recorded incident cancer but were reported to have died with a functioning graft attributed to cancer (n=95) were excluded from this study.

### Data collection

Baseline characteristics recorded by ANZDATA registry included recipient age, gender, race, body mass index, waiting time pre-transplant, comorbidities at transplant (diabetes, coronary artery disease, peripheral vascular disease and cerebrovascular disease) and cause of end-stage kidney disease (ESKD); donor age and type; immunological characteristics included number of human leukocyte antigen (HLA)-mismatches; transplant-related factors such as total ischaemic time (in hours), use of induction therapy, era and initial immunosuppressive agents.

### Ascertainment of cancers

The ANZDATA registry records incident cancers of all kidney transplant recipients. Cancers reported are coded for sites and cell types adapted from the International Classification of Disease for Oncology. It has been demonstrated that the cancer records within ANZDATA registry are accurate, with a high concordance rate when compared to those reported to the New South Wales Cancer Registry, a mandatory reporting requirement within New South Wales [20].

### Clinical outcomes

The primary outcomes of this study included overall graft loss and DCGL. Secondary outcomes included death with a functioning graft, all-cause mortality and cancer-specific mortality.

### Statistical analyses

Data were expressed as number (proportion), mean±standard deviation (SD) and median and IQR where appropriate. Comparisons of baseline characteristics between cancer groups were made by chi-square test and analysis of variance (ANOVA) for categorical and continuous variables, respectively. The associations between incident cancer and outcomes were examined using the adjusted Cox proportional hazard regression analyses, with incident cancer considered as a time-varying covariate in all analyses. Covariates associated with each clinical outcome with p-values of <0.10 in the unadjusted analyses were included in the multivariable-adjusted analyses, although era, donor and recipient age were included because of their likely biological relationship with outcomes. Results were expressed as HR with 95%CI. Site-specific cancer Cox regression analyses were also conducted to assess the relationship between cancer types and death with a functioning graft (with time to event from cancer diagnosis). Sensitivity analysis excluding incident cancers that had occurred within the first 2 years after transplant was undertaken. All analyses were undertaken using SPSS V10 statistical software program (SPSS Inc., North Sydney, Australia) and STATA (version 11 StataCorp LP, College Station, TX).

## SUPPLEMENTARY MATERIALS FIGURES AND TABLES





## Abbreviations

ANOVA: analysis of variance
ANZDATA: Australia and New Zealand dialysis and transplant
CAN/IFTA: chronic allograft nephropathy/interstitial fibrosis and tubular atrophy
CI: confidence interval
CVD: cardiovascular disease
DCGL: death censored graft loss
ESKD: end-stage kidney disease
HLA: human leukocyte antigen
HR: hazard ratio
NMSC: non-melanoma skin cancers
SIR: standardized incidence ratio
SRTR: Scientific Registry of Transplant Recipients
