# Supplementary material for: Long-term allograft and patient outcomes of kidney transplant recipients with and without incident cancer – a population cohort study
Source: Oncotarget. 2017 Sep 8;8(44):77771–82. doi: 10.18632/oncotarget.20781 (PMC5652814; doi:10.18632/oncotarget.20781)
Supplement: Supplementary file 1 [file oncotarget-08-77771-s001.pdf]

Long-term allograft and patient outcomes of kidney transplant recipients with and without incident cancer – a population cohort study

SUPPLEMENTARY MATERIALS

Supplementary Table 1 : Characteristics of the common incident cancers occurring before graft loss.

See Supplementary File 1

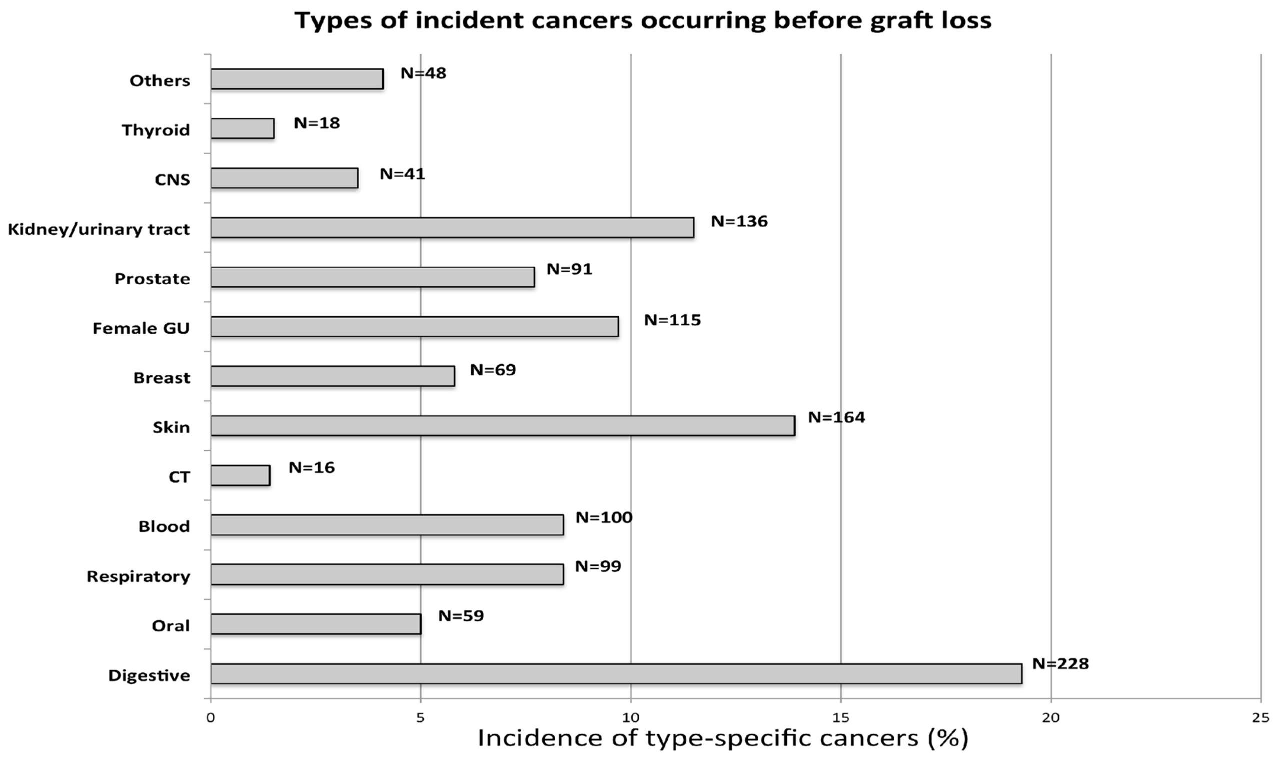

Supplementary Figure 1: The incidence of site-specific cancer types in kidney transplant recipients who have developed cancer before graft loss. CNS – central nervous system, female GT – female genital tract, CT – connective tissue.

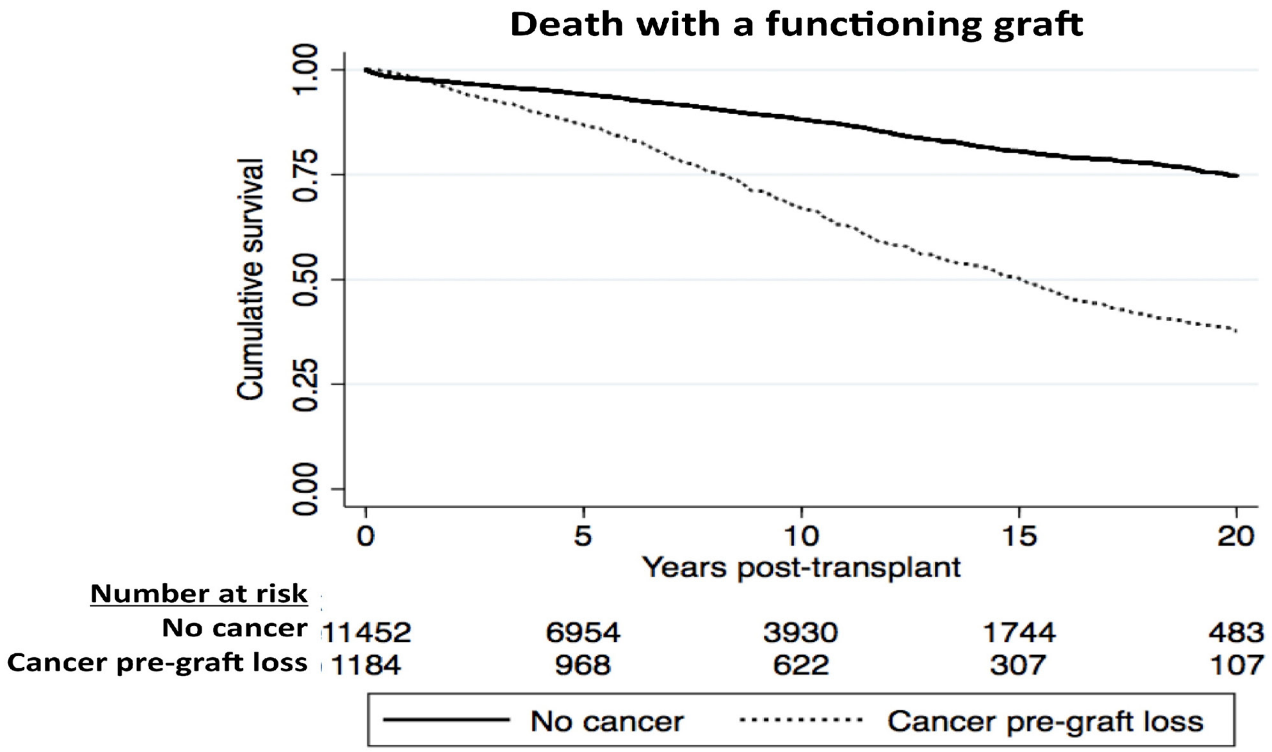

Supplementary Figure 2: Kaplan Meier survival curves with number at risk tables for death with functioning graft of recipients with and without incident cancer. Log-rank $p<0.01$.
