# Supplementary material for: Long-term allograft and patient outcomes of kidney transplant recipients with and without incident cancer – a population cohort study
Source: Oncotarget. 2017 Sep 8;8(44):77771–82. doi: 10.18632/oncotarget.20781 (PMC5652814; doi:10.18632/oncotarget.20781)
Supplement: Supplementary file 2 [file oncotarget-08-77771-s002.docx]

**Supplementary Table 1:**  Characteristics of the common incident cancers occurring before graft loss

|  | Digestive (n=228) | Oral (n=59) | Respiratory (n=99) |
| --- | --- | --- | --- |
| Site of cancer (n) | Colon (85)  Stomach (27)  Oesophagus (16)  Pancreas (16)  Liver (16)  Anal (11) | Lip (9)  Parotid (8)  Tongue (9)  Tonsils (13)  Pharynx (7)  Salivary glands (3) | Lung (75)  Pleura (8)  Nasal (4)  Mediastinal (3) |
| Timing of cancer after transplant (n, %):  0-1 year  >1-3 years  >3-5 years  >5-8 years  >8 years | 28 (12.3)  23 (10.0)  28 (12.3)  51 (22.4)  98 (43.0) | 5 (8.5)  9 (15.3)  8 (13.6)  17 (28.8)  20 (33.8) | 6 (6.1)  19 (19.2)  19 (19.2)  22 (22.2)  33 (33.3) |
| Stage of cancer (n, %):  Unknown  In-situ  Invasive  Regional lymph nodes  Metastasis | 8 (3.5)  44 (19.3)  86 (37.7)  25 (11.0)  65 (28.5) | 4 (6.8)  29 (49.2)  21 (35.6)  3 (5.1)  2 (3.3) | 5 (5.1)  20 (20.2)  24 (24.2)  14 (14.1)  36 (36.4) |
| Overall graft loss (n, %)  Causes (n):  Death with functioning graft  CAN/IFTA  Acute rejection  Withdrawal from cancer (rejection)  Withdrawal from infection (rejection)  Recurrent/de novo glomerulonephritis | 167 (73.2)  142  18  1  1  1  2 | 34 (57.6)  27  6  1  0  0  0 | 84 (84.8)  82  2  0  0  0  0 |
| Death with functioning graft (n, %)  Causes (n):  Cancer  Withdrawal from cancer  Cardiac  Infection | 142 (62.3)  120  2  7  5 | 27 (45.8)  18  0  3  3 | 82 (82.8)  73  1  2  1 |
|  | **Blood (n=100)** | **Skin (n=164)** | **Breast (n=69)** |
| Site of cancer (n) | Bone marrow (41)  Lymph nodes (57)  Spleen (2) | - | - |
| Timing of cancer after transplant (n, %):  0-1 year  >1-3 years  >3-5 years  >5-8 years  >8 years | 9 (9.0)  10 (10.0)  15 (15.0)  24 (24.0)  42 (42.0) | 23 (14.2)  38 (23.3)  29 (17.8)  33 (20.2)  40 (24.5) | 2 (2.9)  14 (20.3)  6 (8.7)  14 (20.3)  33 (47.8) |
| Stage of cancer (n, %):  Unknown  In-situ  Invasive  Regional lymph nodes  Metastasis | 1 (1.0)  10 (10.0)  60 (60.0)  12 (12.0)  17 (17.0) | 1 (0.6)  93 (56.7)  54 (32.9)  6 (3.7)  10 (6.1) | 0 (0.0)  19 (27.9)  32 (47.1)  14 (20.6)  3 (4.4) |
| Overall graft loss (n, %)  Causes (n):  Death with functioning graft  CAN/IFTA  Acute rejection  Withdrawal from cancer (rejection)  Withdrawal from infection (rejection)  Recurrent/de novo glomerulonephritis | 65 (65.0)  55  6  0  1  0  2 | 80 (48.8)  58  21  0  1  0  0 | 31 (44.9)  21  8  0  1  0  0 |
| Death with functioning graft (n, %)  Causes (n):  Cancer  Withdrawal from cancer  Cardiac  Infection | 55 (55.0)  42  0  6  4 | 58 (35.4)  41  1  3  4 | 21 (30.4)  10  0  4  2 |
|  | **Female genital tract (n=115)** | **Prostate (n=91)** | **Kidney and urinary tract (n=136)** |
| Site of cancer (n) | Cervical (80)  Ovarian (10)  Vulva (9)  Uterine (8)  Endometrium (2) | Prostate (84)  Testicular (5)  Penis (2) | Kidney (69)  Graft (26)  Bladder (37) |
| Timing of cancer after transplant (n, %):  0-1 year  >1-3 years  >3-5 years  >5-8 years  >8 years | 8 (7.0)  38 (33.0)  20 (17.3)  31 (27.0)  18 (15.7) | 5 (5.5)  17 (18.7)  14 (15.4)  13 (14.3)  42 (46.1) | 27 (19.9)  16 (11.8)  15 (11.0)  25 (18.3)  53 (39.0) |
| Stage of cancer (n, %):  Unknown  In-situ  Invasive  Regional lymph nodes  Metastasis | 1 (0.9)  91 (79.0)#  9 (7.8)  1 (0.9)  13 (11.4) | 3 (3.3)  58 (63.7)  22 (24.2)  1 (1.1)  7 (7.7) | 2 (1.5)  81 (59.6)  34 (25.0)  4 (2.9)  15 (11.0) |
| Overall graft loss (n, %)  Causes (n):  Death with functioning graft  CAN/IFTA  Acute rejection  Withdrawal from cancer (rejection)  Withdrawal from infection (rejection)  Recurrent/de novo glomerulonephritis | 55 (47.8)  26  25  0  0  0  1 | 35 (38.5)  25  5  0  1  0  2 | 76 (55.9)  45  12  1  1  0  3 |
| Death with functioning graft (n, %)  Causes (n):  Cancer  Withdrawal from cancer  Cardiac  Infection | 26 (22.6)  19  1  1  2 | 25 (27.5)  19  0  2  0 | 45 (33.1)  33  2  2  3 |

*#Includes CIN I-III. CAN/IFTA – chronic allograft nephropathy/interstitial fibrosis.*
